# Research on Calculation of the IOL Tilt and Decentration Based on Surface Fitting

**DOI:** 10.1155/2013/572530

**Published:** 2013-07-14

**Authors:** Lin Li, Ke Wang, Yan Yan, Xudong Song, Zhicheng Liu

**Affiliations:** ^1^School of Biomedical Engineering, Capital Medical University, Beijing 100069, China; ^2^Beijing Tongren Hospital Affiliated to Capital Medical University, Beijing 100730, China

## Abstract

The tilt and decentration of intraocular lens (IOL) result in defocussing, astigmatism, and wavefront aberration after operation. The objective is to give a method to estimate the tilt and decentration of IOL more accurately. Based on AS-OCT images of twelve eyes from eight cases with subluxation lens after operation, we fitted spherical equation to the data obtained from the images of the anterior and posterior surfaces of the IOL. By the established relationship between IOL tilt (decentration) and the scanned angle, at which a piece of AS-OCT image was taken by the instrument, the IOL tilt and decentration were calculated. IOL tilt angle and decentration of each subject were given. Moreover, the horizontal and vertical tilt was also obtained. Accordingly, the possible errors of IOL tilt and decentration existed in the method employed by AS-OCT instrument. Based on 6–12 pieces of AS-OCT images at different directions, the tilt angle and decentration values were shown, respectively. The method of the surface fitting to the IOL surface can accurately analyze the IOL's location, and six pieces of AS-OCT images at three pairs symmetrical directions are enough to get tilt angle and decentration value of IOL more precisely.

## 1. Introduction

The operations of congenital lens subluxation have mostly implanted posterior chamber intraocular lens (IOL) through scleral suture fixation or capsular tension ring to combine with the capsular bag. Serious IOL dislocation has been controlled, but it is difficult to avoid the tilt and decentration of IOL [1–3]. Therefore, to obtain the reliable tilt and decentration of IOL could play an important role in the evaluation of the different operation schemes and effective treatment of postoperative complications.

Ultrasound biomicroscopy (UBM), Purkinje imaging system, anterior segment optical coherence tomography (AS-OCT), and Scheimpflug imaging system are the methods used for measurements of the IOL tilt and decentration in clinical practice. The Purkinje imaging system is simple, but it depends on the radius of curvature of the IOL surfaces. The III image by Purkinje meter can only show parts of the IOL anterior and posterior surfaces. This can have an effect on the radius of curvature and the results of IOL tilt and decentration [4–7]. By UBM, operators can clearly see whether the IOL has tilt and decentration, but the tilt and decentration cannot be directly measured. Moreover, it is a contact inspection method and the deformation of the eyeball extruded by water in bath cup may affect the measured results [8, 9]. Scheimpflug imaging system can rapidly obtain the IOL tilt and decentration values, but it require pupil dilation, which can cause the deviation of the pupil center. So, the IOL decentration measured by the system should be a distance between the IOL center and the nonreal pupil axis, thus the result measured is not accurate and the method has limitations [6, 10–12].

 AS-OCT is a noninvasive and noncontact imaging method of measuring the IOL tilt and decentration and has been widely used in clinical practice. AS-OCT instrument can scan the eyeball at the rotated axis which is the line (called a baseline) passed through the anterior cornea center and the pupil center, and a lot of images can be scanned by AS-OCT at every scanned angle [13, 14]. According to each piece of scanned images, the IOL tilt and decentration are given by AS-OCT instrument. The maximum value of IOL tilts and decentrations obtained from all the images are taken as the IOL tilt and decentration, respectively. In clinical practice, the IOL tilt degree is an angle between the IOL optical axis and the baseline, and the IOL decentration value is the vertical distance from the IOL center to the baseline [6, 11, 15]. Geometrically, the IOL tilt and decentration are two values independent with images. However, we have found that the IOL tilt and decentration values measured on different images at the same scanned angle were not the same. It is possible to result in errors of IOL tilt and decentration. 

 Due to the fact that IOL surface is similar to the spherical [6, 16], we assumed anterior and posterior surfaces of IOL to be spherical surfaces. The equations of the IOL anterior and posterior surfaces were calculated through the AS-OCT image registration and the surface fitting method, and then the IOL tilt and decentration were calculated.

## 2. Materials and Methods

### 2.1. Image Obtaining

**Figure 1:**
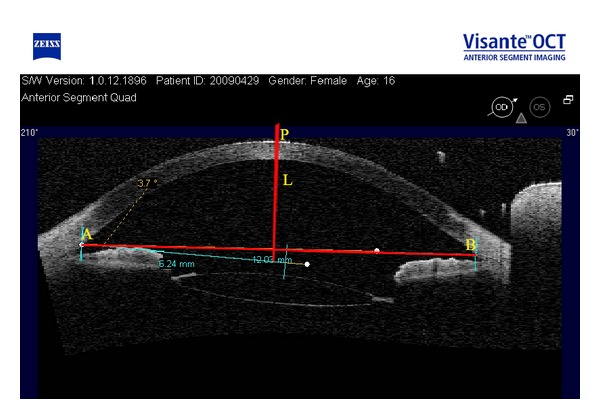
The AS-OCT image at 30 degrees scanned angle. Points A and B are scleral spurs. Point P is the intersection point of perpendicular bisector (L) and corneal anterior surface.

In this study, AS-OCT images of 12 eyes of 8 patients with congenital subluxation lens were provided by the Ophthalmic Research Center of Beijing, Tongren Hospital. The system takes 2000 A scans per second and has an axial resolution of 18 *μ*m and transverse resolution of 60 *μ*m. Moreover, it has a scan speed of 2000 times/second, scan time of 0.125 s/row and frame rate of 30 Hz [9, 13, 14, 17]. For each eyeball, the AS-OCT instrument provides 2-3 pieces of images at a degree from 0° to 180° with periods of 15°, Figure 1 shows a piece of these images. Image format is RGB and image size is 816∗553.

### 2.2. Image Registration

**Figure 2:**
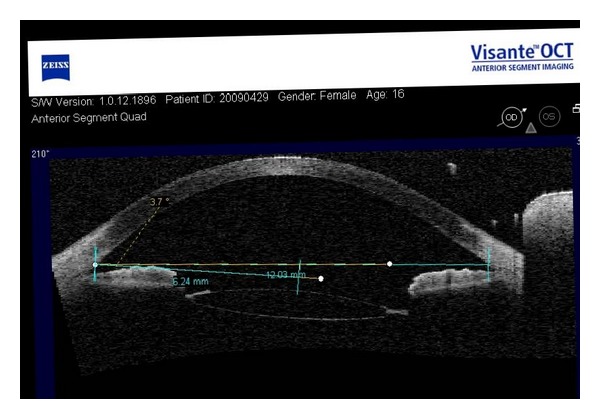
The registered image at 30 degrees scanned angle.

Because the center locations of the corneal anterior surface may be different among images, we need to use image registration for all the images from each scanned angle. The center of the corneal anterior surface is regarded as a reference point defined by the intersection point of corneal anterior surface and perpendicular bisector of the line joining scleral spurs, which is also regarded as reference line (Figure 1). Each picture can be made rigid transformation.  Figure 2 gives one piece of registered images. After image transformation, the pixel values of the center of the corneal anterior surface in images were read to verify the correctness and validity of the rigid body transformation image again.

### 2.3. Collecting IOL Surface Data

From each piece of AS-OCT images, we can obtain two-dimensional pixel coordinates of points of the IOL anterior and posterior surfaces. In order to obtain the three-dimensional coordinates of points of the IOL surface data, Cartesian coordinate system is needed. We set the horizontal direction (alar to zygomatic direction) parallel to *X* axis, the vertical direction (nasal bone to the jaw bone direction) parallel to the *Y* axis, and the ocular axial is set as the *Z* axis. The center of the corneal anterior surface is as the origin of coordinates. Assuming that the two-dimensional coordinate of point A is (*x*, *y*), and the three-dimensional coordinate is (*x*′, *y*′, *z*′), the relations between them are as follows:

(1) $$\begin{array}{c} x^{\prime }=x\cos\alpha ,\;\;y^{\prime }=x\sin\alpha ,\;\;z^{\prime }=y, \end{array}$$

where *α* is scanned angle of AS-OCT.

### 2.4. The Tilt and Decentration of IOL Calculated by Geometric Method

**Figure 3:**
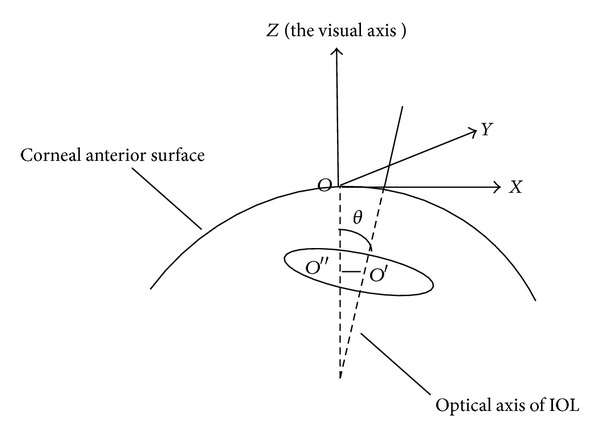
The geometrical definition of IOL tilt and decentration.

From Figure 3, *θ*, known as tilt of IOL, is an angle between visual axis and optical axis of IOL. *β*, defined as tilt at the horizontal direction, is an angle between optical axis of IOL and *X*-axis. *γ*, defined as tilt at vertical direction, is an angle between optical axis of IOL and *Y* axis, and we know the following

(2) cos⁡θ=|c2−c1|rd, cos⁡β=|a2−a1|rd,cos⁡γ=|b2−b1|rd, rd=(a2−a1)2+(b2−b1)2+(c2−c1)2.  

Decentration equation is as follows:

(3) d=d12+[(b2−b1a2−a1)d1+a2b1−a1b2a2−a1]2,d1=12((a2−a1)2(r12−r22)rd2+a2+a1),

where (*a*_1_, *b*_1_, *c*_1_) and (*a*_2_, *b*_2_, *c*_2_) are the coordinates of the spherical center of the fitted IOL anterior and posterior surface with radius of *r*_1_ and *r*_2_, respectively.

### 2.5. IOL Tilt Calculated When the Different Scanned Angles

The IOL tilt and decentration at different scanned angles were calculated based on the equations of the IOL anterior and posterior surface. For the AS-OCT image with scanned angle of *α*, one can get the IOL tilt, denoted by *ω*(*α*), from the following:

(4) $$\begin{array}{c} \cos\omega \left(\alpha \right)=\frac{\left|c_{2}-c_{1}\right|}{\sqrt{r_{d}^{2}-{\left(\left(b_{2}-b_{1}\right)\cos\alpha -\left(a_{2}-a_{1}\right)\sin\alpha \right)}^{2}}}. \end{array}$$

Apparently, as ((*b*_2_−*b*_1_)cos⁡*α*−(*a*_2_−*a*_1_)sin*α*)^2^ = 0, cos⁡*ω*(*α*_0_) = |*c*_2_ − *c*_1_|/*r*_*d*_ attains its minimum value, and *ω*(*α*_0_) is a maxima, which is exactly equal to tilt. Therefore, the tilt (*θ*) should take the maximum value from the tilts calculated at the different scanned angles.

**Figure 4:**
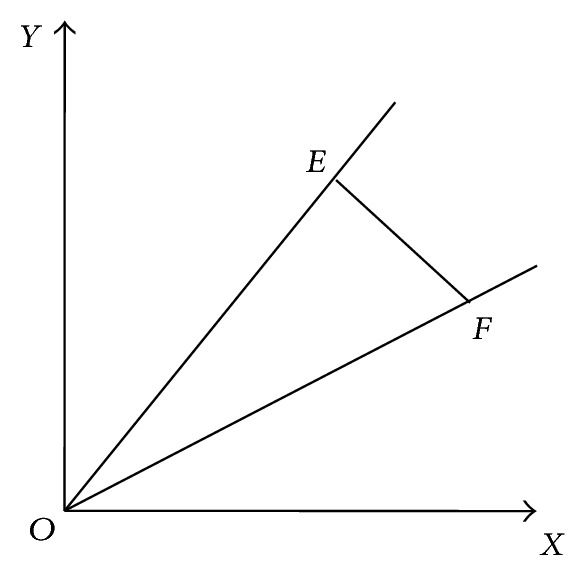
The projection of IOL center point in the plane at different scanned angles.

 For a piece of AS-OCT image with scanned angle of *α* (Figure 4), point *E* is the projection point of image center of IOL, and point F is the projection point of real center of IOL on *XOY* plane (coordinate plane, Figure 3). If there were not any decentration, three points *O*, *E*, and *F* were the same one. The length of segment *OF* is the IOL decentration, denoted by *d*. If there were not any IOL tilt, we know *EF*⊥*OE*. Defining, *α* = *∠* *X* *OE*, *ρ* = *∠* *X* *OF*, and the equation of line *OE* is *y* = *x*tan*α*, for the AS-OCT image with scanned angle of *α*, the IOL decentration, denoted by *d*_2_(*α*), can be obtained

(5) $$\begin{array}{c} d_{2}\left(\alpha \right)=\cos\left(\alpha -\rho \right)d. \end{array}$$

The IOL decentration *d*_2_(*α*) attains its maximum value, the IOL decentration *d*, as long as cos⁡(*α* − *ρ*) = 1. Therefore, the IOL decentration *d* is the maximum value of decentration *d*_2_(*α*) calculated from all of different scanned angle images. If there is a tilt of IOL, segments *EF* and *OE* are nearly orthogonal; then the maximum of *d*_2_(*α*) is close to the IOL decentration *d*.

## 3. Results

**Figure 5:**
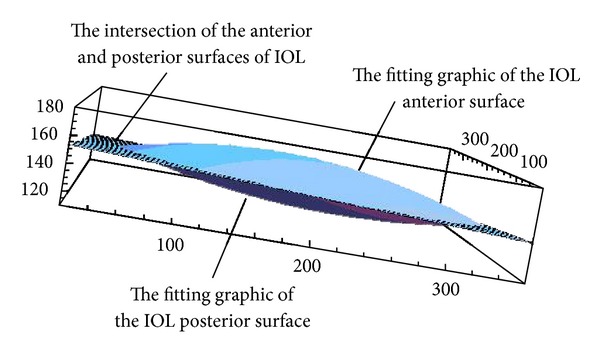
The image of IOL fitting surface and intersection of the anterior and posterior surfaces of IOL.

Figure 5 shows the IOL fitting surface of case  11.  Table 1 gives the IOL tilt and decentration calculated by the surface fitting approach and their values given by AS-OCT instrument. The eighth column of Table 1 gives the diameter of IOL computed by the method of surface fitting. The goodness-of-fit (correlation coefficient *R* ^2^), shown in the ninth-tenth column of Table 1, was between 0.85 and 0.97.

**Figure 6:**
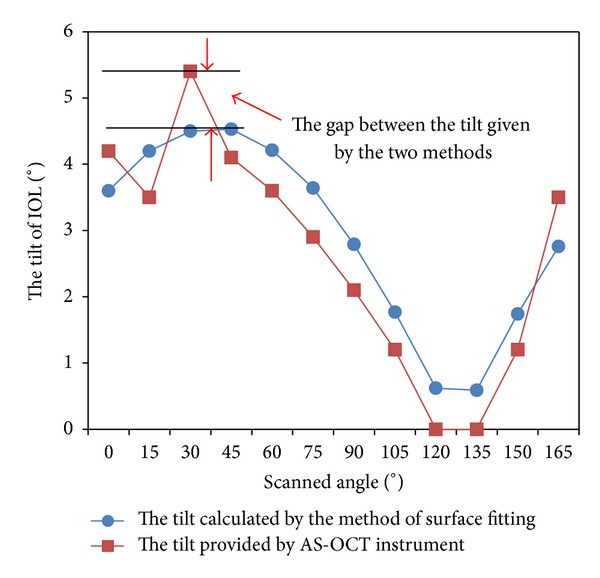
The relation with IOL tilts by the method of surface fitting and AS-OCT and scanned angles.

Figure 6 gives the graph of the tilt *ω*(*α*) defined by formula (4) and provided by AS-OCT instrument for case  12. It is found that the trend of two curves is identical with respect to the change of scanned angle. But the data provided by AS-OCT instrument oscillates with a small amplitude. It definitely affects the estimation of tilt. 

**Figure 7:**
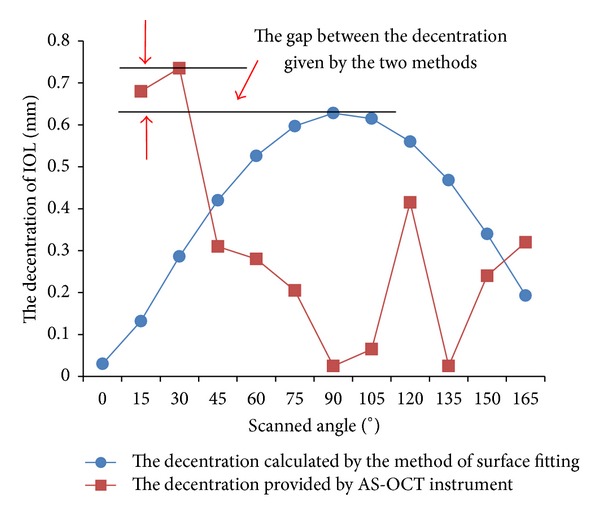
The relation with IOL decentration by the method of surface fitting and AS-OCT and scanned angles.

Figure 7 gives data of the decentration calculated by the method of surface fitting and provided by AS-OCT instrument for case  12. It is shown that the trend of two curves is inconsistent with respect to the change of scanned angle. But the data provided by AS-OCT instrument oscillates with a large amplitude. According to formula (5), the decentration *d*_2_(*α*) changes in accordance with cosine approximately. Clearly, the change of decentration *d*_2_(*α*) calculated by our method is in accordance with cosine, but the decentration *d*_2_(*α*) data provided by AS-OCT instrument is inconsistent with the previous trend.

Table 2 gives the results of the IOL tilt and decentration of three cases by two methods in different scanned angles (The “—” shown in the Table 2 means that this data is not available). The results of IOL tilt and decentration shown in Table 3 were calculated by surface fitting method based on 6, 7, 8, 9, 10, and 11 pieces of AS-OCT images, respectively.

## 4. Discussion

According to AS-OCT images, the IOL tilt and decentration were calculated by collecting data of IOL surface and spherical fitting to the data of IOL anterior and posterior surfaces. The goodness-of-fit (*R* ^2^) is between 0.85 and 0.97.

Korynta et al. [18] showed that the drift and oblique astigmatism can be caused by the IOL decentration being more than 1 mm and the IOL tilt being more than 5 degrees. In our results, the decentration of one eye was greater than 1 mm and the tilts of 3 eyes were greater than 5 degrees. The IOL tilt and decentration of the other 8 eyes basically were within the normal range given by Korynta. It is noticed that most of the cases used in this study are successful in operations. This verified that our results were in accordance with results of the literatures and clinical. In addition, optical diameter of the IOL provided by the manufacturer is 5.5 mm–6 mm. The IOL optical diameter obtained in this study was basically in this range (Table 1). We noticed that the calculated IOL diameters are slightly larger than those provided by the manufacturer. The reason is that the edge of IOL was made with a smooth and certain thickness shape, but we have not taken into account this issue in the calculation (Figure 7). 

The results of tilt calculated by surface fitting (Table 1, 1.76–7.52 degrees) were higher than those of Dhivya's study (0.04–3.6 degrees) [19]. Besides sample differences, the main reason is that the maximum value obtained from the four images (0 degrees, 90 degrees, 45 degrees, and 135 degrees [19]) is possibly less than the IOL tilt (see, (4)). Our results are consistent with those of Xue et al. (tilt, 5.48 ± 1.95 degrees; decentration 1.09 ± 0.65 mm) [20] and Baumeister et al. (tilt, 0.91–6.83 degrees; decentration 0.06 mm–0.51 mm) [21]. In fact, the IOL tilt and decentration were possibly involved in the difference of individuals, surgeons, and measurements.

Assume that IOL anterior and posterior surfaces are spheres. We give the formula of tilt *ω*(*α*) and the approximate expression of decentration *d*_2_(*α*) when the scanned angle is *α*. The two relationships display that both of tilt and decentration continuously change in cosine law. The IOL tilt and decentration are the maximum values of the tilt *ω*(*α*) and decentration *d*_2_(*α*), respectively. However, the tilt and decentration given by AS-OCT instrument are the maximum value of *ω*(*α*) and *d*_2_(*α*) among of all scanned images, respectively. Figures 6 and 7 show that the errors of tilt and decentration produced by AS-OCT are large. In fact, each image contains errors. If these errors are not eliminated, the tilt *ω*(*α*) and decentration *d*_2_(*α*) calculated from each image could cause larger accumulation errors. In our method, the errors are eliminated by registration of images at different scanned directions and selecting as many as possible pixel points to a certain extent. So, the IOL tilt and decentration calculated with the method of surface fitting are accurate.

In order to reduce the pressure of clinical work, it is very significant to provide the method for calculating the more accurate tilt and decentration of IOL and saving the workload. Table 3 gives the results of IOL tilt and decentration calculated with 11, 10, 9, 8, 7, 6 AS-OCT images, respectively by the method. This problem is one of innovations of this study. Although the number of images used to obtain IOL tilt and decentration are 2 (90 degrees and 180 degrees) [6, 15], 4 (0 degrees, 45 degrees, 90 degrees, and 135 degrees) [19], and 5 (five different directions) [22], their results are not verified. Table 3 shows that the IOL tilt and decentration become smaller with the decreasing of the number of images. The tilt and decentration changed in the range of 1 degree and 0.05 mm, respectively. If the errors were neglected in clinical practice. It is suggested that the method can calculate the more accurate tilt and decentration of IOL with scanned angles at the six symmetrical directions.

The IOL tilt measured by AS-COT instrument is the angle between the IOL optical axis and the line joining the centers of the anterior cornea and the pupil. Our method can not only give the IOL tilt, but also the tilt at the horizontal direction and the tilt at the vertical direction. To the knowledge of the authors, it has not been reported whether the horizontal and vertical angles can affect the recovery of vision and optical imaging quality. This may be related to the current methods in clinic leaving two angles unknown. Therefore, it should be studied that whether the horizontal and vertical angles have an impact on visual acuity and visual effect in future.

This study is limited in the following: because IOL was covered by iris and opaque tissues, the middle part of IOL is only displayed in the image, and the point near the IOL boundary cannot be obtained. In addition, artificial selection and obtaining pixel coordinates will bring the errors. This will affect the similarity of the fitting surface and the actual IOL surface. Therefore, it should be studied that the method can reduce errors in collecting data for improving the fitting goodness in future. In addition, this study assumed that the IOL surface was spherical. But the aspheric surface cannot be thought about. It is hoped that the IOL surface can be calculated with the method of aspheric fitting in future. Moreover, compared with two methods of spherical and aspheric fitting, the results calculated with two methods can be more close to the real values in clinical. 

In conclusion, the method of the surface fitting to the IOL surface can accurately analyze the IOL's location, and six piece of AS-OCT images at three pairs symmetrical directions are enough to get tilt angle and decentration value of IOL more precisely.

## Figures and Tables

**Table 1:** The IOL tilt and decentration obtained by the method of surface fitting.

Case	The method of AS-OCT instrument		The method of surface fitting						
	Tilt/°	Decentration/ mm	The IOL tilt (θ)/°	The tilt at horizontal direction/°	The tilt at vertical direction/°	Decentration/ mm	Optical diameter/ mm	*R* ^2^ (IOL anterior surface)	*R* ^2^ (IOL posterior surface)
1	7.90	1.01	7.52	86.19	83.53	0.88	5.82	0.67	0.81
2	4.30	0.83	3.35	87.82	87.46	0.71	5.91	0.89	0.91
3	3.30	0.73	2.13	89.30	87.98	0.29	5.87	0.87	0.93
4	4.90	0.85	2.79	87.80	89.13	0.80	5.81	0.85	0.86
5	6.90	0.67	5.86	88.04	84.47	0.28	5.77	0.86	0.86
6	4.20	2.33	3.92	86.23	88.94	1.08	5.85	0.86	0.83
7	3.60	0.77	1.76	88.59	88.94	0.67	6.12	0.93	0.88
8	3.30	0.51	2.2	88.18	88.77	0.45	5.91	0.83	0.89
9	5.40	0.78	2.13	88.24	88.80	0.76	5.96	0.90	0.85
10	5.20	0.73	4.08	87.85	86.54	0.27	6.15	0.97	0.87
11	6.80	0.71	5.85	89.07	84.21	0.44	6.21	0.91	0.85
12	5.40	0.74	4.55	86.40	87.22	0.63	6.06	0.93	0.83

**Table 2:** IOL decentration and tilt obtained from the image with different scanned angle.

Scanned angle/°	Tilt/°						Decentration/mm					
	Case 6		Case 4		Case 12		Case 6		Case 4		Case 12	
	Surface fitting	AS-OCT	Surface fitting	AS-OCT	Surface fitting	AS-OCT	Surface fitting	AS-OCT	Surface fitting	AS-OCT	Surface fitting	AS-OCT
0	2.72	3.60	2.21	2.50	3.60	4.20	0.18	1.86	0.15	0.01	0.03	—
15	3.93	1.50	1.70	3.40	4.20	3.50	0.68	1.69	0.35	0.85	0.13	0.68
30	3.82	1.90	1.47	4.10	4.50	5.40	0.44	1.39	0.52	0.79	0.29	0.74
45	3.41	3.20	1.21	2.00	4.53	4.10	0.17	1.32	0.66	0.78	0.42	0.31
60	2.83	1.30	0.40	0.50	4.21	3.60	0.11	2.40	0.75	0.33	0.53	0.28
75	2.03	4.20	0.30	0.50	3.64	2.90	0.38	1.67	0.80	0.67	0.60	0.21
90	1.1	2.00	0.92	1.60	2.79	2.10	0.63	2.16	0.78	0.16	0.63	0.03
105	0.04	0.00	1.40	1.00	1.77	1.20	0.83	2.34	0.72	0.59	0.62	0.07
120	0.98	2.40	1.85	2.10	0.62	0.00	0.98	2.34	0.61	0.31	0.56	0.42
135	1.13	3.10	2.21	2.70	0.59	0.00	0.87	—	0.45	0.03	0.47	0.03
150	2.74	2.40	2.40	3.50	1.74	1.20	1.07	1.94	0.26	0.03	0.34	0.24
165	3.38	2.30	2.35	4.90	2.76	3.50	1.01	1.77	0.06	0.77	0.20	0.32
IOL	3.92	4.20	2.37	4.90	4.55	5.40	1.08	2.40	0.80	0.85	0.63	0.74

**Table 3:** IOL tilt and decentration calculated by surface fitting method based on 6, 7, 8, 9, 10, and 11 pieces of AS-OCT images.

AS-OCT image	Case 5		Case 6		Case 1		Case 4	
	Tilt/°	Decentration/mm	Tilt/°	Decentration/mm	Tilt/°	Decentration/mm	Tilt/°	Decentration/mm
11	5.86	0.28	3.92	1.08	—	—	—	—
10	6.10	0.25	3.94	1.02	7.50	0.88	2.79	0.80
9	6.05	0.27	3.73	1.03	7.85	0.81	2.56	0.79
8	5.79	0.25	3.76	1.03	7.49	0.84	2.54	0.81
7	6.45	0.21	3.70	1.02	6.69	0.86	2.63	0.79
6	6.22	0.24	3.68	1.03	6.35	0.82	2.54	0.79
